# Overexpression of lncRNAs with endogenous lengths and functions using a lncRNA delivery system based on transposon

**DOI:** 10.1186/s12951-021-01044-7

**Published:** 2021-10-02

**Authors:** Yin Zhang, Yong-Xin Huang, Xin Jin, Jie Chen, Li Peng, Dan-Lan Wang, Yun Li, Xin-Yi Yao, Jian-You Liao, Jie-Hua He, KaiShun Hu, Daning Lu, Yabin Guo, Dong Yin

**Affiliations:** 1 Guangdong Provincial Key Laboratory of Malignant Tumor Epigenetics and Gene Regulation, Research Center of Medicine, Sun Yat-Sen Memorial Hospital, Sun Yat-Sen University, Guangzhou, 510120 People’s Republic of China; 2 Guangdong-Hong Kong Joint Laboratory for RNA Medicine, Sun Yat-Sen Memorial Hospital, Sun Yat-Sen University, Guangzhou, 510120 People’s Republic of China

**Keywords:** lncRNA, Non-viral delivery, Ectopic overexpression, Sleeping Beauty transposon, Lentivirus

## Abstract

**Background:**

Long noncoding RNAs (lncRNAs) play important roles in many physiological and pathological processes, this indicates that lncRNAs can serve as potential targets for gene therapy. Stable expression is a fundamental technology in the study of lncRNAs. The lentivirus is one of the most widely used delivery systems for stable expression. However, it was initially designed for mRNAs, and the applicability of lentiviral vectors for lncRNAs is largely unknown.

**Results:**

We found that the lentiviral vector produces lncRNAs with improper termination, appending an extra fragment of ~ 2 kb to the 3ʹ-end. Consequently, the secondary structures were changed, the RNA–protein interactions were blocked, and the functions were impaired in certain lncRNAs, which indicated that lentiviral vectors are not ideal delivery systems of lncRNAs. Here, we developed a novel lncRNA delivery method called the Expression of LncRNAs with Endogenous Characteristics using the Transposon System (ELECTS). By inserting a termination signal after the lncRNA sequence, ELECTS produces transcripts without 3ʹ-flanking sequences and retains the native features and function of lncRNAs, which cannot be achieved by lentiviral vectors. Moreover, ELECTS presents no potential risk of infection for the operators and it takes much less time. ELECTS provides a reliable, convenient, safe, and efficient delivery method for stable expression of lncRNAs.

**Conclusions:**

Our study demonstrated that improper transcriptional termination from lentiviral vectors have fundamental effects on molecular action and cellular function of lncRNAs. The ELECTS system developed in this study will provide a convenient and reliable method for the lncRNA study.

**Graphic Abstract:**

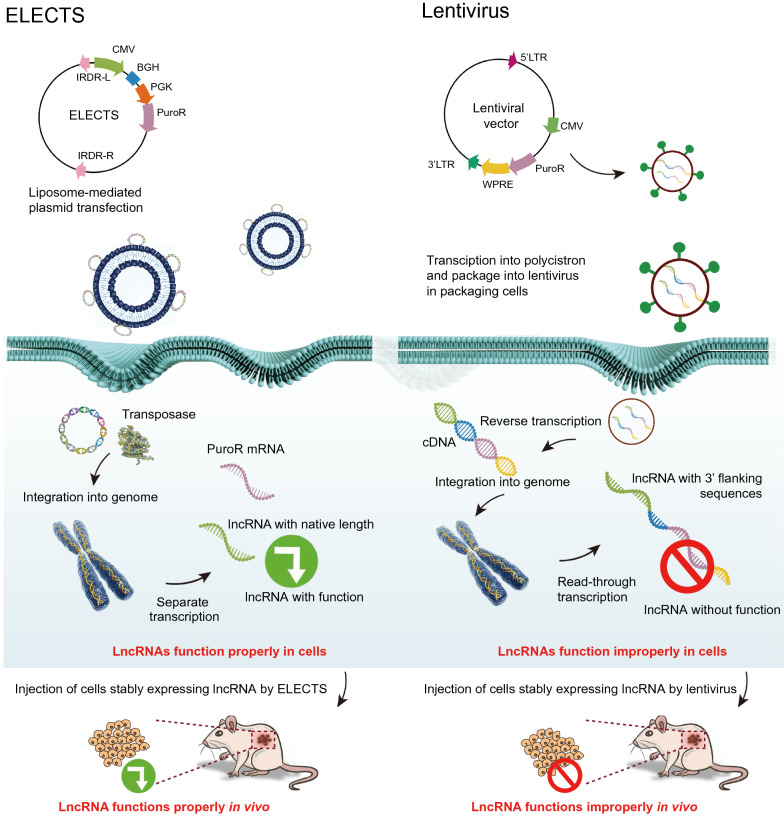

**Supplementary Information:**

The online version contains supplementary material available at 10.1186/s12951-021-01044-7.

## Background

Long noncoding RNAs (lncRNAs) are a type of RNA which are longer than 200 nucleotides with no apparent protein-coding potentials. By interacting with protein, DNA and RNA, lncRNAs function as important regulators in a variety of biological and pathological processes [1–3]. With the development of high-throughput sequencing, more and more lncRNAs have been identified, yet only a small group of them have been functionally studied [4]. Delivery of lncRNAs with various nanomaterials provide new strategies for cancer therapy [5–8].

Lentivirus is the most commonly used tool for stable expression of lncRNAs. As initially designed for protein coding genes, complex 3ʹ-flanking sequences adjacent to the cloning site were designed for efficient virus particle production and gene expression. Moreover, it is reported that certain transcripts from LTR-retrotransposons/retroviruses can also function like lncRNAs [9–11], which may bring more side effects for lncRNA delivery by lentivirus. Since the mRNA translation terminates at the stop codon, the 3ʹ-flanking sequences of the vector attached to the target sequence have little affect the protein products. In contrast, lncRNAs play roles at RNA level, the primary sequence, length and secondary structure are all critical factors for the properties of lncRNAs [12]. However, as a potential problem, whether the 3ʹ-flanking sequences affect the lncRNA functions has long been overlooked [13, 14]. Here, using traditional lentiviral vectors, we overexpressed six lncRNAs with validated function. Surprisingly, four of them failed to reproduce the phenotypes reported previously. Further investigation of HCCL5 and HOTAIRM1, showed that lncRNAs expressed by lentiviral vectors are transcribed together with flanking lentiviral elements, resulting in much longer (> 2 kb) transcripts than their endogenous versions. These results indicated that lentivirus may be unsuitable for certain lncRNAs. The flanking lentiviral elements can alter the RNA secondary structure, reduce the RNA–protein interactions and impair the functions of lncRNAs. Since transcription and reverse transcription are essential for the propagation of retroviruses, transcriptional termination signals cannot be inserted between the two long terminal repeats (LTRs). Thus, the precisely transcriptional termination of lncRNA cannot be achieved by lentiviral vectors.

DNA transposon is another ideal system for stable expression and gene therapy [15]. Unlike retroviruses, DNA transposon vectors do not need transcription for their integration, which greatly facilitates the integration of plasmid into the host genome [16]. Here, we developed a novel non-viral delivery system called Expression of LncRNAs with Endogenous Characteristics using the Transposon System (ELECTS), which expresses lncRNAs with no flanking sequences and retains almost the same length as the endogenous lncRNAs. Moreover, using liposome or other nanoparticles to deliver lncRNAs, ELECTS does not need virus packaging or infection processes. On one hand, there is no potential risk of infection for the operators, on the other hand, it is more time efficient than the lentiviral vectors. It takes only 1 week for ELECTS, while about 2 weeks for lentivirus to successfully obtain stable expression cells (Table 1). Thus, our ELECTS offers a reliable, safe and efficient way for stable expression of lncRNAs.

**Table 1:** Comparison of ELECTS and other available vectors for stable expression of lncRNAs

vectors	ELECTS	Lentivirus	pcDNA
Integration rate	++++	+++	±
Expression cassette after integration	Intact	Intact	Mostly partial
Transcription termination site	BGH	3ʹLTR	BGH
Length of lncRNA product	As endogenous	Flanked by extra sequences	As endogenous
Secondary structure of lncRNA	Native	May be altered	Native
Function of lncRNA	Normal	May be altered or abolished	Normal
Time	~ 1 week	10–14 days	Up to 6 months
Biosafety	safe	Potential risk	safe

Our study demonstrated that improper transcriptional termination from lentiviral vectors has a fundamental effect on molecular action and cellular function of lncRNAs, which is of great importance but frequently overlooked by the researchers. Our study will provide a convenient and reliable stable expression system for the community of lncRNA researchers.

## Materials and methods

### Cell culture, transfection, nanomaterials characterization and stable cell line establishment

Hepatocellular carcinoma cell lines HepG2, PLC, SK-Hep1, colorectal cancer cell line SW480 and lentivirus packaging cell HEK293T were obtained from American Type Culture Collection (ATCC) and maintained in Dulbecco’s Modified Eagle Medium (DMEM, Gibco, 11995065) containing 10% fetal bovine serum (Gibco) and 1% penicillin–streptomycin (Invitrogen). In this study, for establishing stable cell lines with ELECTS, ELECTS and SB100X (Addgene #34879) plasmids (10:1) were co-transfected into HepG2 and SK-Hep1 cells using lipid nanoparticle Lipofectamine 3000 (Thermo Fisher #L3000015), and the cells were selected by puromycin for ~ 5 days. Transmission electron microscopy (TEM) was conducted. Zeta potential and physicochemical properties of nanomaterials were determined by dynamic light scattering (DLS) with a Malvern Zetasizer, NANO ZS (Malvern Instruments Limited, UK). For the lentiviral vector, pCDH plasmids were co-transfected into HEK293T cell with the packaging plasmids psPAX2 (Addgene #12260) and pMD2.G (Addgene #12259) using polyethylenimine transfection reagent (Polysciences, #23966). Viral particle supernatants were harvested and concentrated using 44% PEG8000 (Sigma-Aldrich, #89510) and 4 M NaCl. HepG2 and SK-Hep1 cells were infected with the lentiviruses in the presence of 5 ug/mL Polybrene (Yeasen, #40804ES76) and selected by puromycin for 5–7 days.

### Plasmids construction

The ELECTS plasmid was derived from the pSB vector described previously [17]. The CAG promoter and GateWay cassette of the pSB were removed using EcoRV and SphI restriction enzymes, the CMV promoter, Multiple Cloning Site and BGH sequences were amplified from pcDNA3.1 vector and inserted into the pSB using ClonExpress II One Step Cloning Kit (Vazyme Biotech Co., Ltd, #C112). The primers used for constructing ELECTS plasmid were listed in Additional file 2: Table S1. For ELECTS-delGFP, the GFP and IRES sequences were removed using NsiI and BglII restriction enzymes. For ELECTS-delBGH, The BGH sequences were removed using XbaI and PaeI restriction enzymes. The lncRNA HCCL5 (NR_135174.1), HOTAIRM1 (NR_038367.1), LINC00364 (NR_130792.1), PCBP2-OT1 (NR_109828.1), BANCR (NR_047671.2) and GAPLINC (NR_110429.1) fragments were synthesized according to the reference sequences from NCBI, which have been verified by rapid amplification of cDNA ends (RACE) previously [18–20], and inserted into the ELECTS and the pCDH plasmid by NheI and NotI restriction sites. All vectors were validated by Sanger sequencing in IGE BIOTECHNOLOGY LTD.

### Real-time quantitative PCR and 3ʹ RACE

When cells were grown to 80–90% confluence, the total RNA was collected using Trizol Reagent (Invitrogen, #15596026), and then reverse-transcribed using PrimeScript RT reagent Kit (Takara, #RR047A) and analyzed by LightCycler^®^ 96 real-time PCR thermocycler (Roche). The expressions of lncRNAs were normalized to β-actin transcripts. The primers used for PCR were synthesized by IGE BIOTECHNOLOGY LTD and the sequences were listed in Additional file 2: Table S1.

3ʹ RACE assay was conducted using SMARTer RACE Kit (Takara, #634858) following the manufacturer’s instructions. The primers used for 3ʹ RACE were listed in Additional file 2: Table S1. The expected bands of 3’RACE was excised and purified using agarose gel purification kit (TIANGEN, #DP214) and cloned into pMD18-T vector (Takara, #6011) for Sanger sequencing.

### Western blot

Cells were lysed by RIPA buffer, supplemented with proteinase inhibitor cocktail (Bimake, #B14001) and phosphatase inhibitor cocktail (Bimake, #B15001). Protein quantification was determined by Bradford. The protein lysates were subjected to Western blot assay described previously [3], with the following primary antibodies specific to E-cadherin (1:1000; Cell Signaling Technology, #3195P), N-cadherin (1:1000; Cell Signaling Technology, #13116P), CCNA1 (1:1000; BD Biosciences, #611268), CCNB1 (1:1000; BD Biosciences, #554178), CCND1 (1:1000; BD Biosciences, #556470), and GAPDH (1:4000; Bioss, #bs-2188R).

### Northern blot

The Northern blot was conducted using DIG Northern Starter Kit (Roche, #12039672910) according to the manufacturer’s instructions. 10 µg of total RNA was separated in a 1% formaldehyde agarose gel and transferred onto Immobilon-Ny+ membrane (Millipore, #INYC00010). The primers used for preparation probes were listed in Additional file 2: Table S1.

### RNA immunoprecipitation (RIP) assay

RIP was performed as previously reported [2]. Briefly, cells were first crosslinked with 0.3% formaldehyde and then added to glycine at a concentration of 0.125 M to stop the crosslink. Cells were lysed with RIPA buffer supplied with protease and RNase inhibitors. The cell lysate was incubated with SUZ12 antibody (ABclonal, #A7786) or control IgG (Cell Signaling Technology, #2729) at 4 °C for 2 h and then incubated with protein G Dynabeads (Invitrogen, #10004D) at 4 °C overnight. The beads were then washed and digested with proteinase K. RNA samples were purified by phenol chloroform extraction and ready for RT-qPCR assay.

### RNA pull-down assays

The short and long RNA products were synthesized using a TranscriptAid T7 High Yield Transcription Kit (Thermo Fisher, #K0441). The RNAs were labeled with biotin using a Pierce™ RNA 3ʹ End Desthiobiotinylation Kit (Thermo Fisher, #20163) and then purified with a GeneJET RNA Purification Kit (Thermo Fisher, #K0731). The lncRNAs pull down assays were conducted using a Pierce™ Magnetic RNA–Protein Pull-Down Kit (Thermo Fisher #20164) following the manufacturer’s instructions. The lysate of Hela cells was incubated with biotin labeled RNAs. The proteins were analyzed with a Western Blot using SUZ12 antibody (Cell Signaling Technology, #3737).

### Cell proliferation assays and cell cycle analysis

MTT (3-[4,5-dimethylthiazol-2-yl]-2,5 diphenyl tetrazolium bromide) assays were performed as described previously. 2000 SK-Hep1 cells and 2500 HepG2 cells were plated in 96-well plates in 100 μl media containing 10% FBS, and cultured for 5 days. For colony formation assay, 800 SK-Hep1 cells and 1000 HepG2 cells were plated in 6-well plates (Jet Bio-Filtration Co., Ltd) for about 2 weeks, then the colonies were fixed using 4% paraformaldehyde and stained with 0.1% crystal violet for 30 min. The colony numbers were counted using Image J software. EdU assays were performed by a Cell-Light EdU Apollo567 In Vitro Kit (RIBOBIO, C10310) and imaging using inverted fluorescence microscope, For cell cycle assay, indicated cells were digested and washed with PBS and then fixed in 70% ethanol at 4 °C overnight, then incubated with RNase A and propidium iodide (PI, Beyotime, #ST511) for 10 min and analyzed by flow cytometry. All assays were performed in triplicate and repetition.

### Xenograft in nude mice model

SK-Hep1 cells (2 × 10^6^) stably expressing HOTAIRM1 using pCDH and ELECTS respectively were subcutaneously injected under the skin of six 5-week-old female nude mice. Tumor volumes were measured (length × width^2^ × 0.5) and evaluated every 3 days. After 26 days, mice were sacrificed, and the tumors were excised and their weight was evaluated. This animal study was approved by Laboratory Animal Center of Sun Yat-sen University.

### Statistical analysis

Student's *t* test was used for statistical analysis, *p* values of less than 0.05 were considered statistically significant.

## Results

### The performance of lentiviral vector for lncRNA expression

**Fig. 1:**
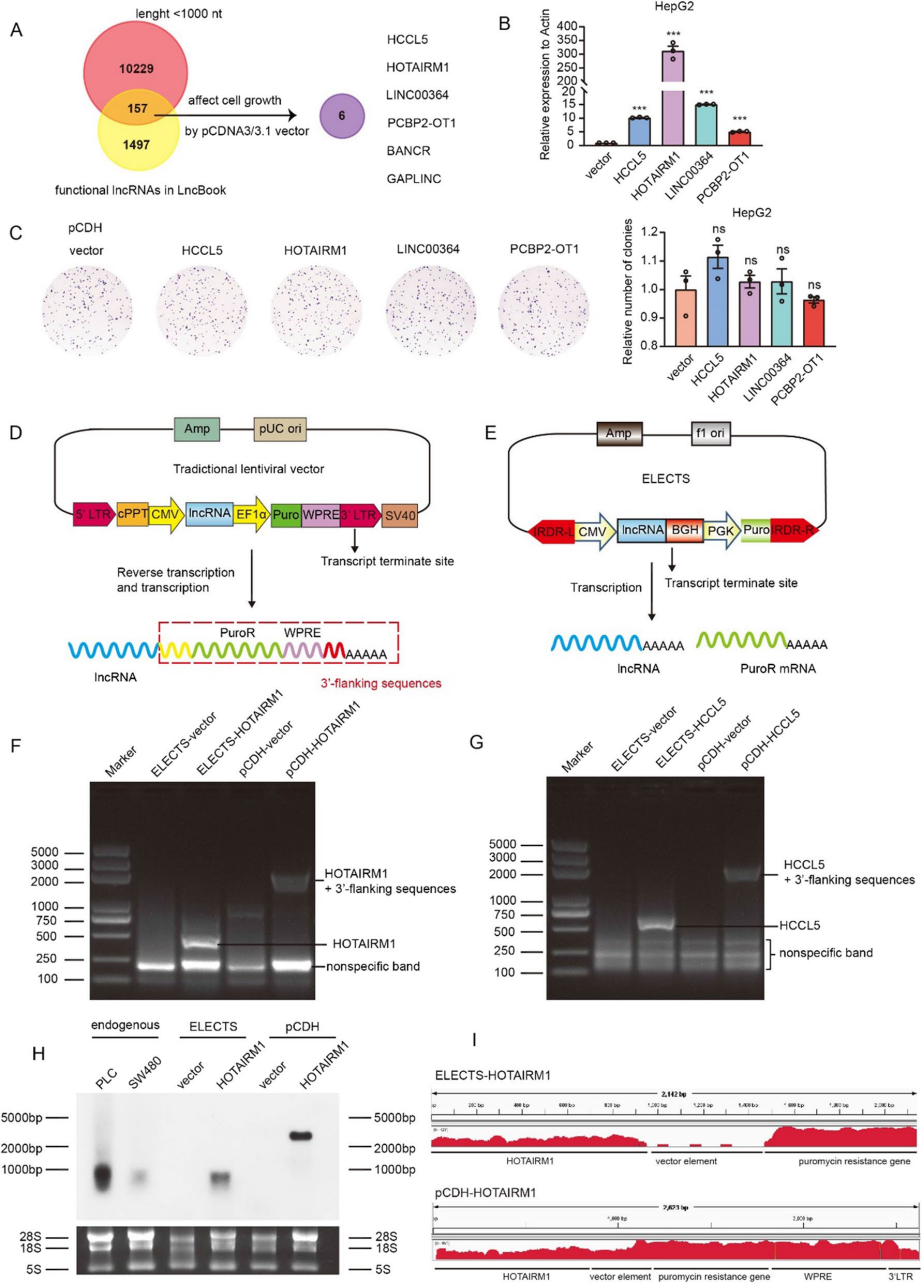
Lentiviral expression vectors affect the function of some lncRNAs. **A** Screen for lncRNAs whose function may be affected when overexpression with lentivirus vectors. **B** The expression levels of lncRNAs expressed by pCDH in HepG2 cells were tested using qRT-PCR. **C** The effects of lncRNAs expressed by pCDH on the cell proliferation were tested using colony formation assay in HepG2 cells. **D** A simplified schematic diagram shows the mechanism of lentivirus vector pCDH in transcriptional termination of exogenous expressed lncRNAs. **E** A simplified principle of transcriptional termination of exogenous expressed lncRNAs of ELECTS. **F**, **G** 3ʹ RACE showed the lengths of HOTAIRM1 (**F**) and HCCL5 (**G**) transcribed using ELECTS and pCDH respectively. **H** Northern Blot revealed the lengths of the exogenous HOTAIRM1 products expressed using ELECTS and pCDH. **I** RNA-seq results showing the signal of HOTAIRM1 transcripts produced by ELECTS (top panel) and pCDH (bottom panel), the reads were mapped to the corresponding vector sequences

Since transcription and reverse transcription are required for replication and integration of the lentivirus, transcriptional termination signals or poly (A) sites were absent after the destination gene [21, 22]. Theoretically, the lncRNA could be transcribed together with all the downstream elements into a long and complex transcript, which may affect the function of lncRNAs. To test this assumption, we selected six lncRNAs based on the following criteria: (1) shorter than 1000 nt, because we think lncRNAs with less nucleotides are more likely influenced by the flanking sequences; (2) have been proven to influence cell proliferation using the pCDNA3/3.1 vector (Fig. 1A). We expressed these lncRNAs in HepG2 cells using pCDH, a widely used commercial lentiviral vector. The expression levels of the lncRNAs were evaluated by qRT-PCR assays (Fig. 1B). Surprisingly, despite all of these lncRNAs having been successfully overexpressed, four of them showed no significant change of cell proliferation measured by colony formation assays (Fig. 1C).

### Design of a method that allows stable expression of lncRNAs with endogenous features

The transcriptional termination signal or poly (A) site could separate lncRNA transcript from vector elements [21]. However, as mentioned above**,** a transcriptional termination signal or a poly (A) site will disrupt the reverse transcription of the entire viral RNA and dramatically reduce the titer of the virus [21, 22], which is a dilemma for expressing lncRNAs by lentiviral vector.

To solve the problem raised by the lentiviral vector, we attempted to develop a tool for stable expression of lncRNAs using transposon vectors. We employ the pSB plasmid, a SB transposon that was used in our previous study [17, 23], to construct a plasmid for overexpressing lncRNAs. The original CAG promoter of the pSB plasmid contains the first exon and the first intron of the chicken beta-actin gene, which will be transcribed with the destination sequence as part of 5ʹ-flanking sequences, so we replaced the CAG promoter with the CMV promoter. We also removed the chloramphenicol resistance gene and Gateway cassette from the vector (Additional file 1: Fig. S1A). Then we inserted a Multiple Cloning Site (MCS) followed by a bovine growth hormone polyadenylation signal (BGH polyA) [24], which can efficiently terminate the transcription right after lncRNA sequences (Fig. 1E). The transcriptions of the GFP and puromycin resistance genes are driven by a PGK promoter independent of the transcription of the lncRNA. We also expressed these selected lncRNAs in HepG2 cells using ELECTS and tested the expression levels of the lncRNAs by qRT-PCR assays (Additional file 1: Fig. S1B). Colony formation assays showed that the exogenous lncRNAs expressed by ELECTS promoted cell growth, but lncRNAs expressed by pCDH did not (Additional file 1: Fig. S1C). While GFP is useful for evaluating the transfection efficiency (Additional file 1: Fig. S1D) and xenograft tumors, it may influence the application of FISH or immunofluorescence. Therefore, we constructed two versions of ELECTS plasmids, one with GFP and one without (ELECTS-delGFP).

**Scheme 1.**
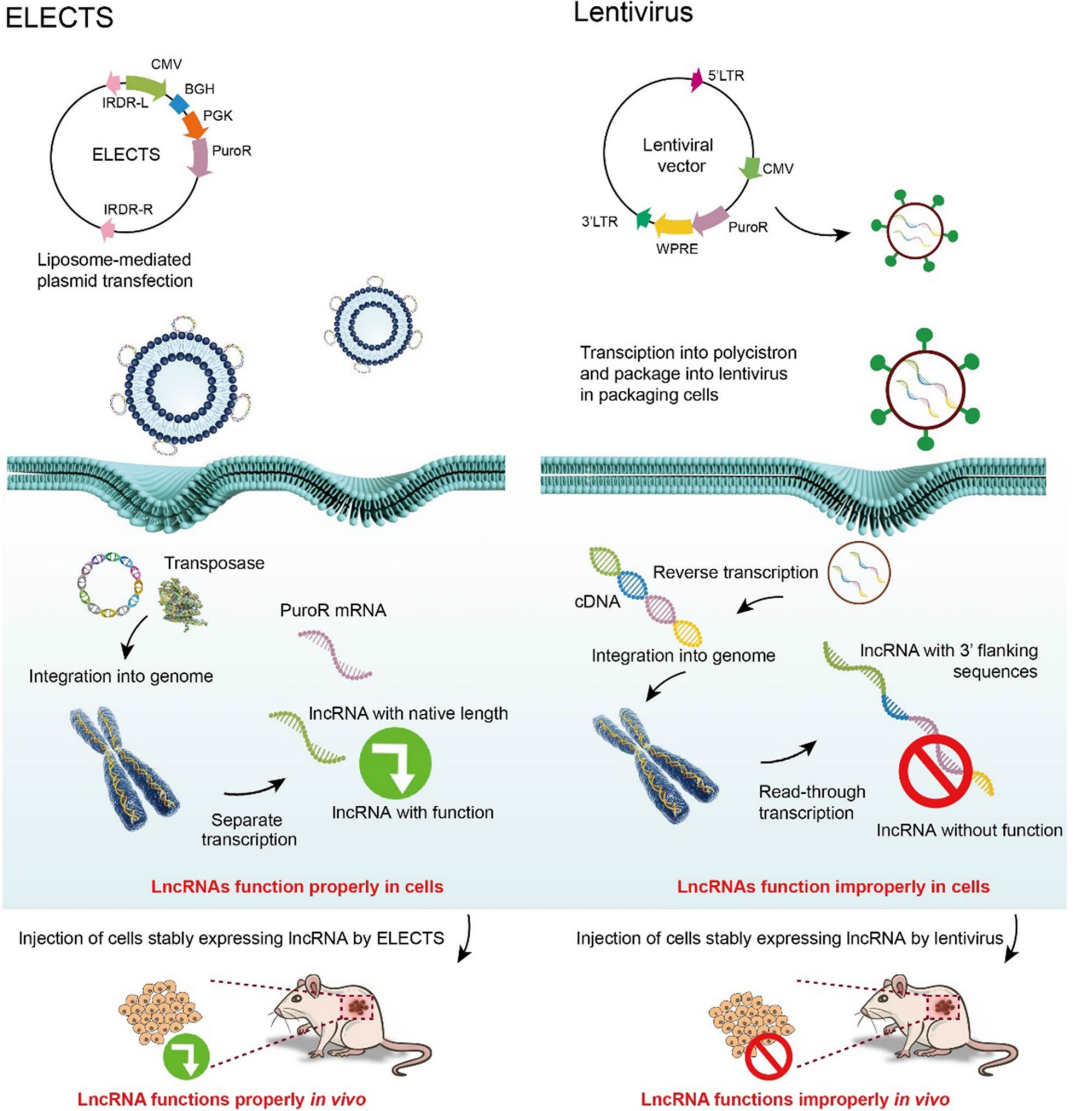
Schematic diagram of ELECTS and lentiviral vector exogenous expression mechanism. ELECTS non-viral vector was loaded with liposome nanoparticles and directly delivered into destination cells. The lentiviral vector should be packaged in 293 T cells and then the viral nanoparticles were used to infect destination cells. LncRNAs expressed by ELECTS function properly both in vivo and in vitro while lncRNAs expressed by lentiviral vectors may function improperly

Thus, in the ELECTS, the transcription of the lncRNA gene is initiated by the CMV promoter and terminated by the BGH poly (A) signal, and the selection marker cassette is driven by an independent PGK promoter. All the elements above are flanked by the IRDR-L/R of SB transposon, which is recognized by the SB transposase (Scheme 1). When co-transfected with SB100X expression plasmid, the cassette between the SB IRDR-L/R will be cleaved and integrated into the chromosomes of the host cell, resulting in stable expression of lncRNAs. The ELECTS can also be co-transfected with SB11 expression plasmid (Addgene, #26552), a less active transposase, to achieve a moderate over-expression (Additional file 1: Fig. S1E, F).

### Lentiviral vector expresses lncRNAs with 3ʹ-flanking sequences while ELECTS produces lncRNAs with proper termination

We speculated that phenotypic differences induced by pCDH and ELCETS could be due to the different characteristics of exogenous lncRNAs. Lacking termination signal in pCDH may result in extra sequences at 3ʹ end. To test this assumption, we selected 2 lncRNAs from above, HCCL5 and HOTAIRM1, for further investigation. It takes about 2 weeks to successfully obtain stable expression cells using pCDH vectors, but only ~ 1 week using ELECTS vectors, revealing ELECTS to be a timesaving approach (Table 1).

Total RNA of HepG2 cells stably overexpressing lncRNA by the two vectors was isolated, and 3ʹ RACE assays were performed to detect the 3ʹ end sequences of lncRNAs. LncRNAs expressed by ELECTS showed almost the same length of 3ʹ end as expected, while lncRNAs expressed by pCDH exhibited a much larger band than expected (Fig. 1F, G). The sequencing results of 3ʹ RACE revealed only 165 bp of cloning sites and partial BGH sequences were flanked at the 3ʹ end of ELECTS expressed lncRNA (Additional file 3: Table S2). However, the lncRNA expressed by pCDH showed a 1976 bp extra fragment at the 3ʹ end containing cloning sites, EF1α promoter, puromycin resistance gene, WPRE and partial LTRs at the 3ʹ end (Additional file 3: Table S2).

To further confirm this phenomenon, we analyzed the HOTAIRM1 transcripts by northern blot. We used two cell lines, PLC and SW480, which have relatively high expression levels of endogenous HOTAIRM1, as a control to the position of endogenous HOTAIRM1 on the gels. The size of ELECTS expressed HOTAIRM1 is ~ 900 bp, almost the same length as endogenous HOTAIRM1, whereas, the HOTAIRM1 produced by pCDH is ~ 2.8 kb, about three to four times as the former (Fig. 1H). What’s more, we performed RNA-seq using HepG2 stable cells. The lncRNA transcripts from pCDH are “read-through”, resulting in a long and improper transcript isoform. While the elements in ELECTS including target gene and selection gene are transcribed separately, the length of the lncRNA transcripts are equal to endogenous ones (Fig. 1I), which is consistent with the northern blot results.

Taken together, this data revealed that ELECTS successfully transcribes lncRNAs that were basically the same length as the endogenous transcripts, while a large stretch of sequence was appended to the lncRNAs by lentiviral vector (Fig. 1D), which is even much longer than the lncRNAs themselves.

### ELECTS retains proliferation-promoting phenotypes of lncRNA that abolished by lentiviral vector in vitro

**Fig. 2:**
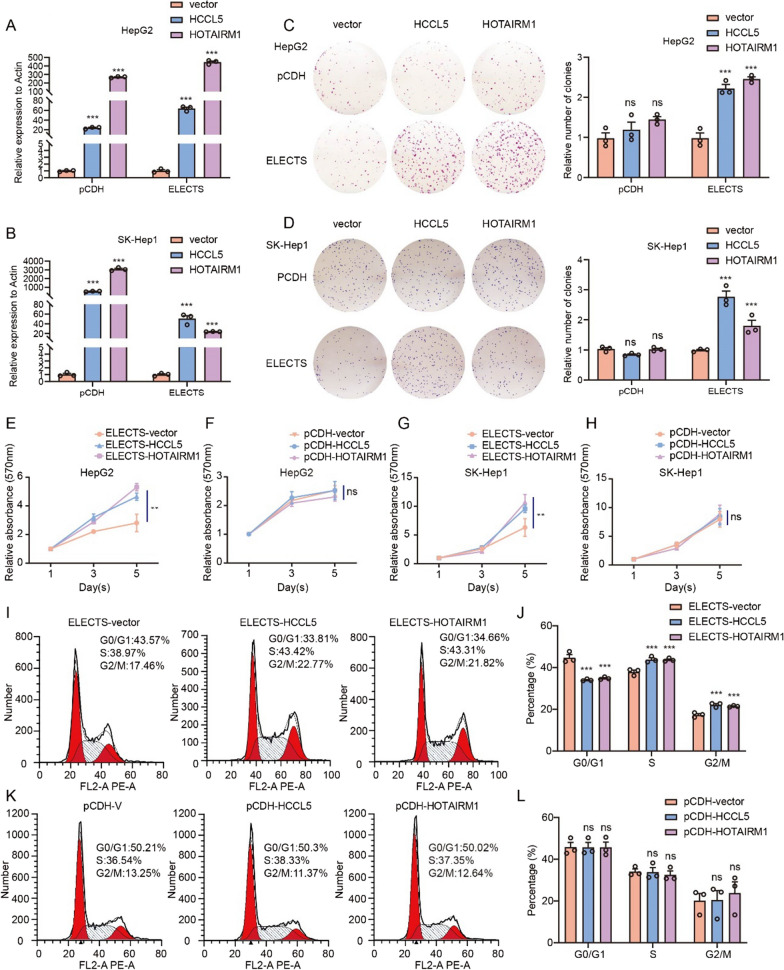
The performance of lentiviral vector and ELECTS for expressing lncRNA HOTAIRM1 and HCCL5. **A**, **B** The expression levels of HOTAIRM1 and HCCL5 by pCDH or ELECTS in HepG2 (**A**) and SK-Hep1 (**B**) cells were tested using qRT-PCR. **C**, **D** The effects of HOTAIRM1 and HCCL5 expressed by pCDH and ELECTS respectively on the cell proliferation were tested using colony formation assay in HepG2 (**C**) and SK-Hep1 (**D**) cells. **E**–**H** MTT assays showed the proliferation effects of HOTAIRM1 and HCCL5 expressed by ELECTS (**E**, **G**) and pCDH (**F**, **H**) respectively in HepG2 (**E**, **F**) and SK-Hep1 (**G**, **H**) cells. **I**–**L** Cell flowmetry assays showed the effects of HOTAIRM1 and HCCL5 expressed by ELECTS (**I**,** J**) and pCDH (**K**, **L**) respectively on proliferation in HepG2 cells

To test the performance of the ELECTS vector in detail, total RNA was isolated and qRT-PCR assays were conducted to evaluate the expression level of the lncRNAs. About 400- and 250-fold upregulation of HOTAIRM1 were observed, using ELECTS and pCDH respectively in HepG2. For HCCL5, the overexpression level was about 50- and 25-fold using ELECTS and pCDH, respectively (Fig. 2A). ELECTS showed a slightly higher expression level than pCDH. While in the SK-Hep1 cell line, the overexpression efficiency of pCDH is slightly higher than ELECTS (Fig. 2B).

HCCL5 is a lncRNA that promotes tumorigenesis in hepatocellular carcinoma and is involved in promoting cell growth and metastasis [18]. HOTAIRM1 functions as an oncogene in multiple types of cancers [19, 20, 25, 26]. The proliferation-promoting function of these lncRNAs have been verified previously by overexpression experiments using pcDNA3/3.1 vectors. To investigate the function of the exogenous lncRNA expressed by different vectors, we assessed the cell proliferation by colony formation and MTT assays. Cells stably over-expressing both HCCL5 and HOTAIRM1 by ELECTS showed significant increase in proliferation, while over-expression of these lncRNAs by pCDH showed no effect on cell growth both by colony formation (Fig. 2C, D) and MTT assays (Fig. 2E–H).

**Fig. 3:**
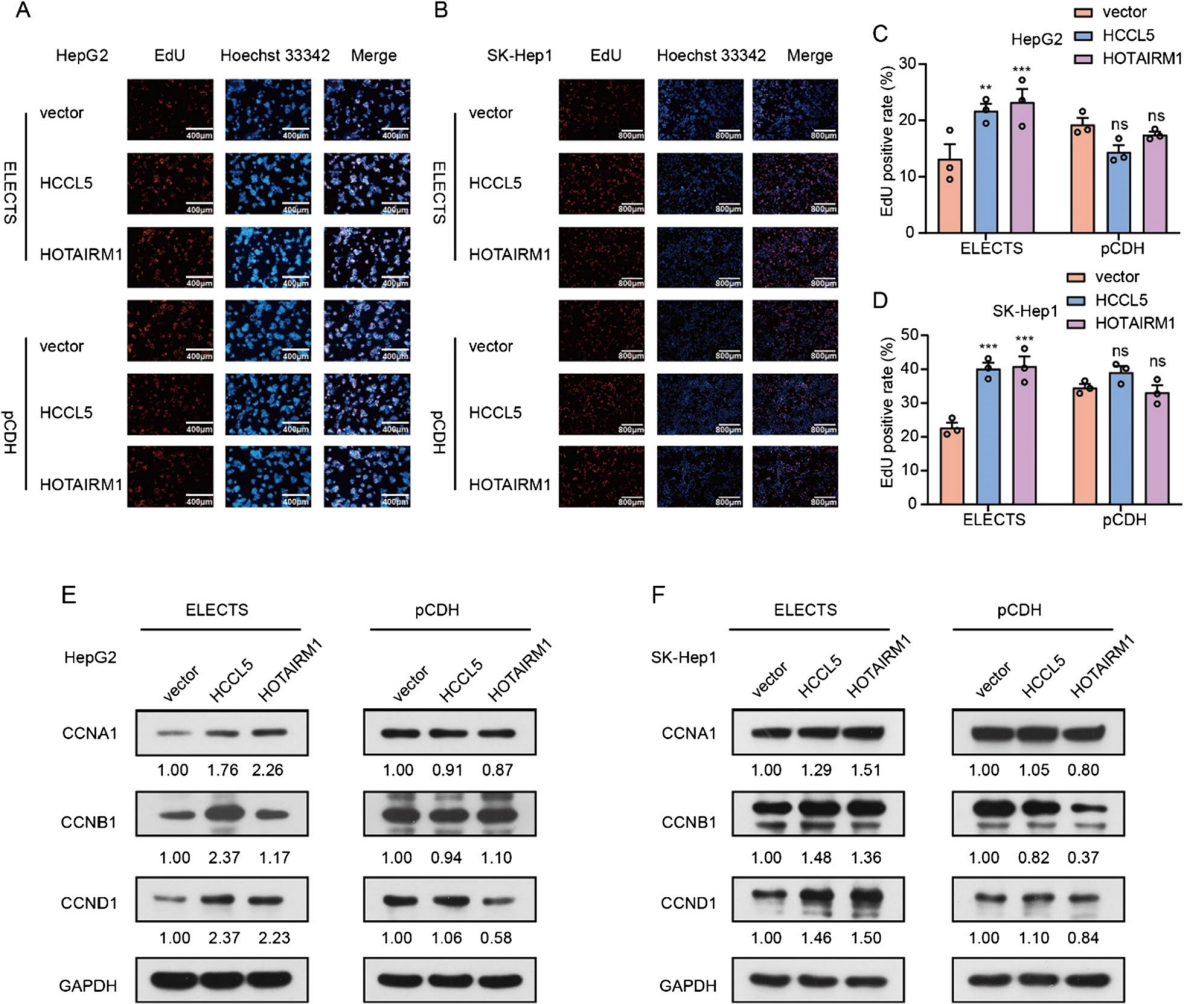
The effects on cell growth of lncRNA HOTAIRM1 and HCCL5 ectopically expressed using pCDH and ELECTS in vitro. **A**, **C** EdU assay showed the differences of proliferation function between exogenous HOTAIRM1 and HCCL5 expressed by pCDH or ELECTS in HepG2 cells. **B**, **D** The effects of HOTAIRM1 and HCCL5 expressed by pCDH and ELECTS respectively on the cell proliferation were tested by EdU assay in SK-Hep1 cells. **E**, **F** Western blot showed the variations of cell cycle-related proteins when expression of lncRNAs using ELECTS and pCDH respectively in HepG2 (**E**) and SK-Hep1 (**F**) cells

We further detected the impact of lncRNAs on cell cycle by flow cytometry, the amount of S phase and G2/M phase cells in HepG2 cells were significantly increased when overexpressed HCCL5 and HOTAIRM1 by ECLECTS, while no significant change of cell was observed when expressing lncRNA with pCDH (Fig. 2I–L). EdU assays were also used to confirm the change of cell proliferation rate by measuring the proportion of cells at S phase. The proportion of S-phase cells was increased in cells overexpressing lncRNA HCCL5 and HOTAIRM1 by ELECTS, but not by pCDH (Fig. 3A–D). We also detected the expression level of the cyclin family, which functions as CDK kinase regulators and regulates progress of cell cycle. CCNA1 regulates the transition of S phase and G2 phase, CCNB1 regulates the G2/M transition, CCND1 regulates G1/S transition. In cells stably expressed HCCL5 and HOTAIRM1 by ELECTS, the expression levels of these proteins are higher than control cells, while no obvious increase of protein levels were observed after these lncRNAs were expressed by lentiviral vector in either HepG2 or SK-Hep1 cells (Fig. 3E, F). These results demonstrated although both lentiviral vector and ELECTS can achieve efficient overexpression of HCCL5 and HOTAIRM1, only ELECTS-expressed lncRNAs have retained functions, lncRNAs with lentiviral failed to reproduce the functions previously observed by pcDNA3/3.1.

### Ectopic HCCL5 expressed by ELECTS rather than lentiviral vector enhances cell migration

**Fig. 4:**
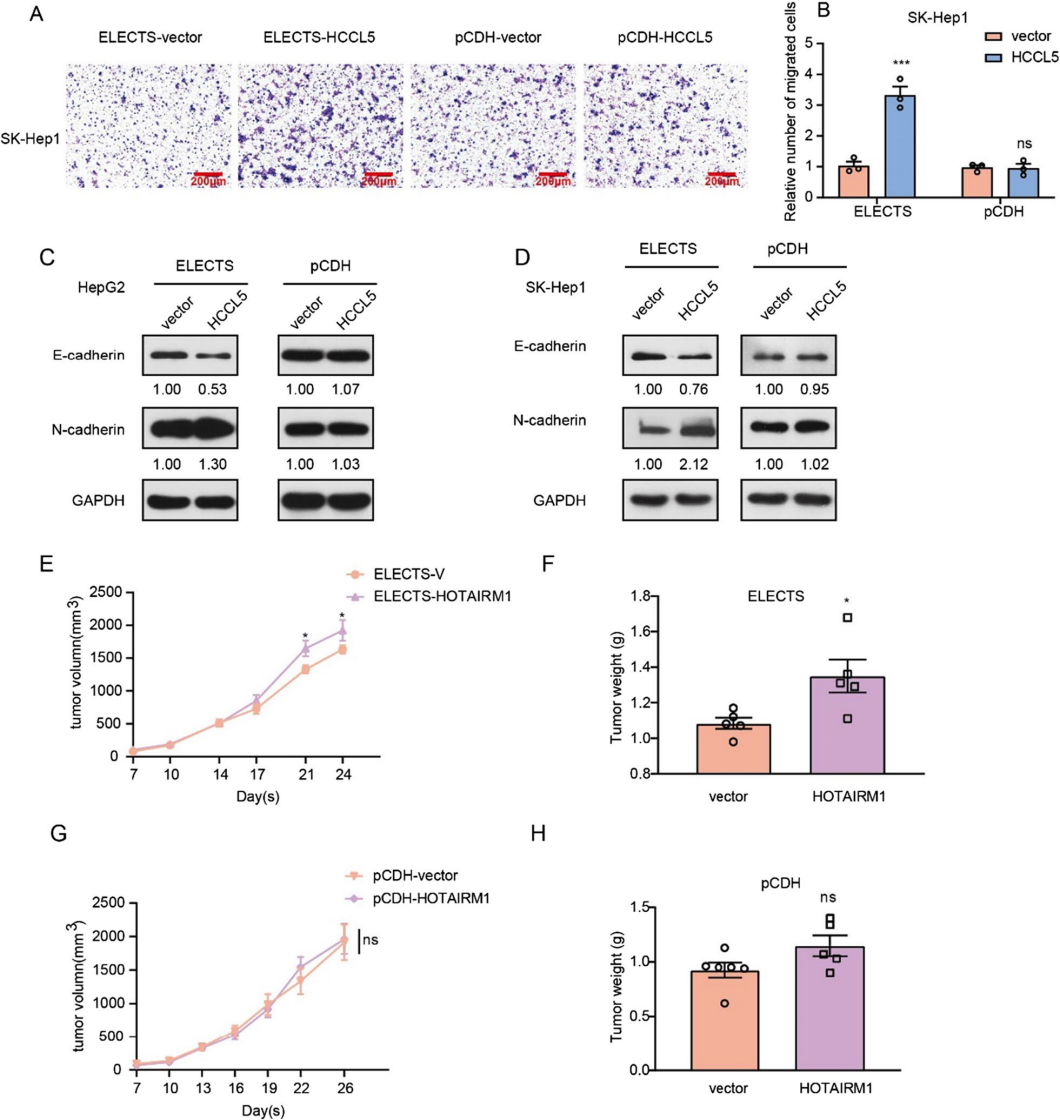
The performance of lncRNA produced by pCDH or ELECTS on cell migration and growth of xenograft tumors. **A**, **B** Transwell assays showed the migration effects of HCCL5 expressed by pCDH and ELECTS respectively in SK-Hep1 cells. **C**, **D** Different effect on EMT markers of exogenous lncRNA HCCL5 expressed by ELECTS (**C**) and pCDH (**D**). **E**, **G** Tumor growth curve of nude mice inoculated with SK-Hep1 cells overexpressing HOTAIRM1 using ELECTS (**E**) and pCDH (**G**) respectively. **F**, **H** Tumor weight of SK-Hep1 cells overexpressing HOTAIRM1 using ELECTS (**F**) and pCDH (**H**) respectively

Previous studies of lncRNA HCCL5 have also revealed that it can function as an oncogene by promoting epithelial–mesenchymal transition (EMT) and metastasis of cancer cells. We further investigated the effect of lncRNA on migration rates by different overexpression systems. Transwell assays showed exotic expression lncRNA HCCL5 by ELECTS can promote cell migration, while cells stably expressed HCCL5 by pCDH showed no advantages of migration (Fig. 4A, B). We also measured the expression level of EMT markers by Western Blot, the downregulated expression level of E-cadherin and upregulated expression level of N-cadherin (Fig. 4C, D) was observed in SK-Hep1 and HepG2 cells overexpressed HCCL5 using ELECTS, but no significant change was found when expressed by the lentiviral vector. These data revealed that lncRNA HCCL5 overexpressed by ELECTS, but not by lentivirus, can significantly promote cell migration in vitro by facilitating the EMT process.

### Ectopic HOTAIRM1 expressed by ELECTS rather than lentiviral vector promotes xenografts growth in vivo

Stable overexpression is usually used for the study of lncRNA functions that need long term observation, one of the most common applications is investigating in vivo function using xenograft tumors. To test whether the exogenous lncRNA expressed by different vector can function in vivo, SK-Hep1 cells stably expressing HOTAIRM1 by ELECTS or lentiviral vector were subcutaneously injected into nude mice to form xenograft tumors. For HOTAIRM1 overexpressed by ELECTS, the tumor volume and weight are significantly higher than the control group (Fig. 4E, F, Additional file 1: Fig S2A). Nevertheless, there are no differences of tumor volume and weight between cells stably expressing HOTAIRM1 and control group when expressed by lentiviral vector (Fig. 4G, H, Additional file 1: Fig S2B).

### Flanking sequences from lentiviral vector influence secondary structure of lncRNAs

**Fig. 5:**
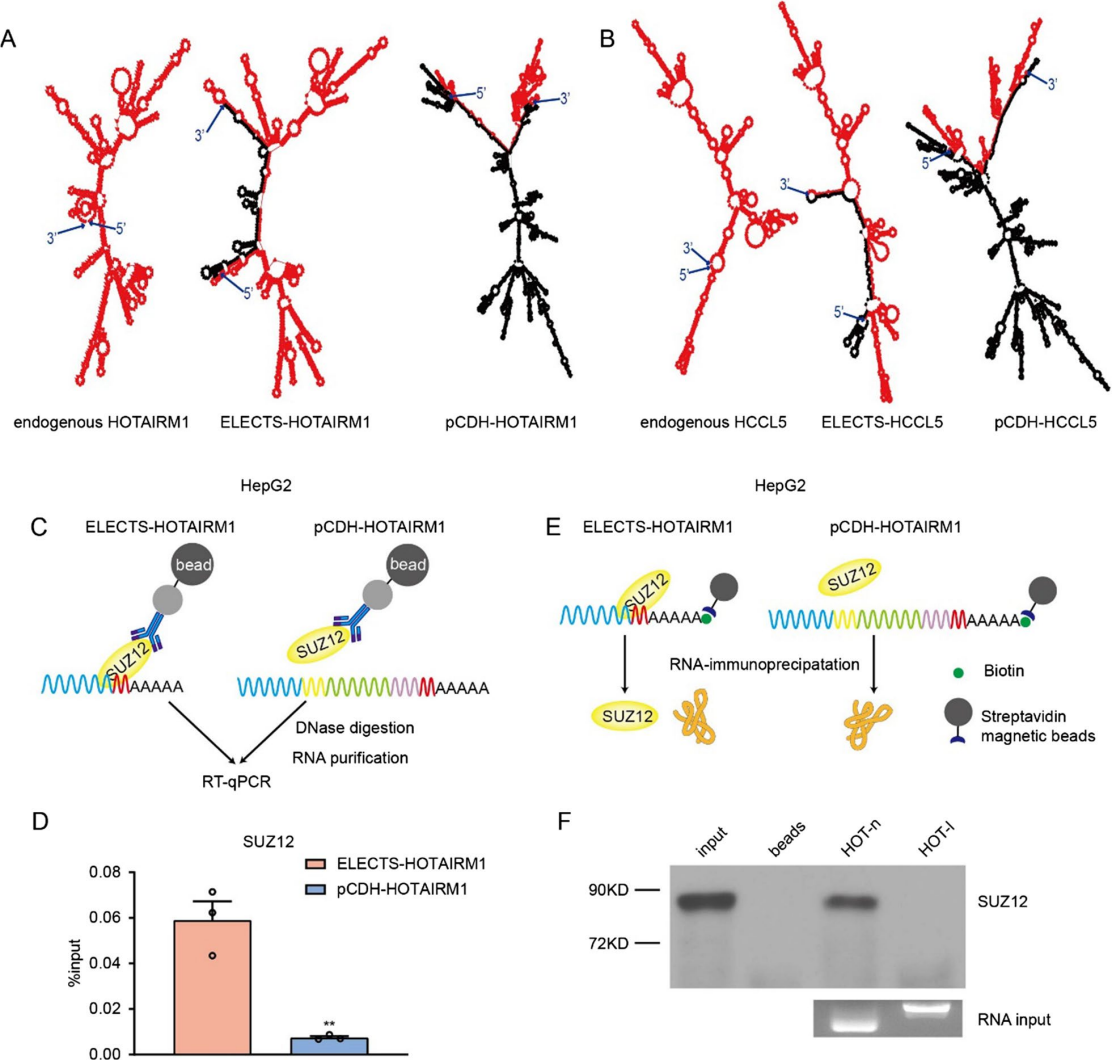
The potential effect on secondary structure and RNA–protein interaction by 3ʹ-flanking sequences. **A**, **B** Comparison of the secondary structures of lncRNA HOTAIRM1 (**A**) and HCCL5 (**B**) among endogenous, ELECTS-produced and pCDH-produced transcripts. The RNA secondary structure was analyzed using the RNAfold web server. **C** Schematic diagram for RIP-qPCR. **D** RIP-qPCR was performed to analyze the enrichment of HOTAIRM1 after immunoprecipitation of SUZ12 in HepG2 cells overexpressing HOTAIRM1, using ELECTS and pCDH respectively. **E** Schematic diagram for RNA pull down. **F** Identification of HOTAIRM1 binding protein SUZ12 was analyzed by RNA pull down followed by western blot in HepG2 cells. HOT-n: native full-length of HOTAIRM1. HOT-l: HOTAIRM1 with extra 3ʹ-flank sequences according to the result of 3ʹ RACE

The secondary structure is critical for the role of lncRNAs, the RNA structure appears to be the main functional unit and tends to be more evolutionarily conservative [1, 27]. To study the potential changes of secondary structures by extra elements from the vectors, we conducted secondary structure analysis using the RNAfold [28], a widely used web server for the prediction of RNA secondary structure. By comparing the predicted structures of native lncRNAs, lncRNAs expressed by ELECTS and pCDH, we found the secondary structures of ELECTS expressed HOTAIRM1 and HCCL5 are basically the same as those of the native transcripts, while lncRNAs expressed by pCDH showed substantially different secondary structure (Fig. 5A, B). The change of structure of pCDH expressed lncRNAs were mainly caused by the abundant base pairs between lncRNAs and flanking sequences from vectors. Moreover, the lentiviral flanking sequences also form complex structures, which can also reshape the overall structures of lncRNAs.

### Flanking sequences from lentiviral vector impair the RNA–protein interaction

Since lncRNAs usually function through interacting with proteins, we next investigated whether the flanking sequences interfere with the interaction between lncRNAs and proteins. HOTAIRM1 has been reported to regulate the expression of certain genes by interacting with SUZ12, a polycomb repressive complex 2 (PRC2) component [29]. To examine the intensity of RNA–protein interactions, cells stably expressing HOTAIRM1 were immunoprecipitated using SUZ12 antibody and the associated RNA were detected by RT-PCR (RIP-qPCR). The HOTAIRM1 expressed by ELECTS showed much higher SUZ12-affinity than that expressed by pCDH (Fig. 5C, D). We further assessed the interaction using RNA pull-down assay. According to the results of 3ʹRACE, different RNAs corresponding to the HOTAIRM1 transcripts produced by ELECTS and pCDH were synthesized in vitro, and equal amounts of biotin-labeled RNAs were incubated with cell lysis. Western Blot showed there was a strong SUZ12 band from proteins pulled-down by the short transcripts, while no SUZ12 was detected from the proteins enriched by the long transcripts (Fig. 5E, F). This data indicated that the flanking sequences introduced by the lentiviral vector impaired the lncRNA-protein interactions.

### The proper termination signal is vital for the function of certain lncRNAs

**Fig. 6:**
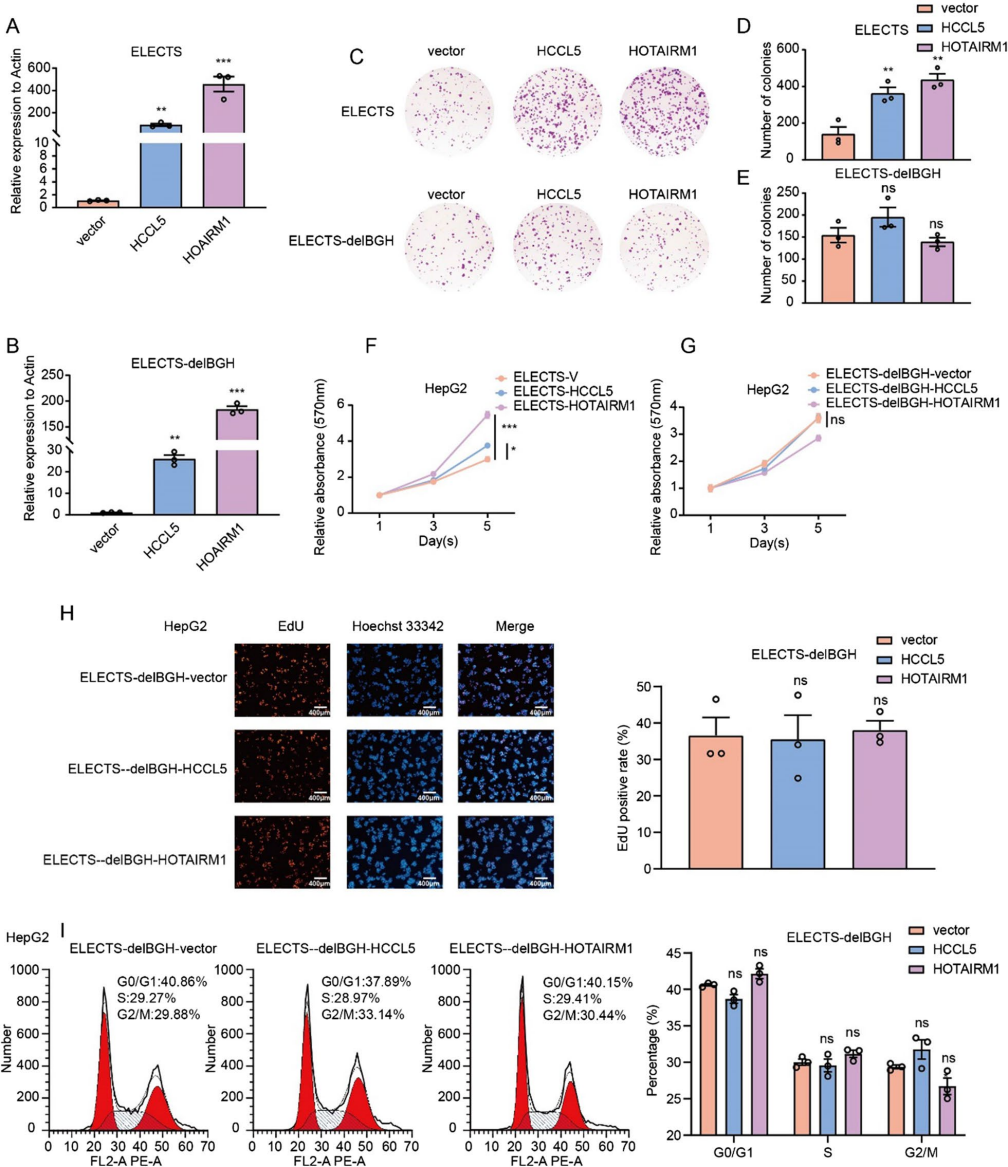
The termination signal is critical for the functions of certain lncRNAs. **A**, **B** qPCR showed the expression levels of HOTAIRM1 and HCCL5 using ELECTS (**A**) and ELECTS-delGFP (**B**) in HepG2 cells. **C**–**E** Colony formation assay showed the effects of exogenous HOTAIRM1 expressed using ELECTS (**D**) and ELECTS-delGFP (**E**) on cell proliferation respectively in HepG2 cells. **F**, **G** MTT assays showed the proliferation effects of HOTAIRM1 and HCCL5 expressed by ELECTS (**E**) and ELECTS-delGFP (**F**) respectively in HepG2 cells. **H** EdU assay showed the differences of proliferation function between exogenous HOTAIRM1 and HCCL5 expressed by ELECTS-delGFP in HepG2 cells. **I** Cell flowmetry assays showed the effects of HOTAIRM1 and HCCL5 expressed by ELECTS-delGFP on proliferation in HepG2 cells

To further evaluate the importance of the proper termination to the lncRNAs. We deleted the BGH sequence that helps efficiently terminate the transcription at the 3ʹ end of the lncRNA sequence from the ELECTS vector, resulting in a new vector designated as ELECTS-delBGH. As expected, northern blot showed that the transcripts produced by ELECTS-delBGH exhibit an additional band that was ~ 2500 bp longer than that produced by ELECTS (Additional file 1: Fig. S2C). The secondary structures of the transcripts were also analyzed using RNAfold as mentioned above, and fundamental change of secondary structure was found when the downstream elements were transcribed with lncRNAs (Additional file 1: Fig. S3). Various assays were conducted to examine the function of these exogenous lncRNAs. Although ELECTS-delBGH, like the lentiviral vector, can efficiently express the lncRNAs (Fig. 6A, B), colony formation assay (Fig. 6C–E), MTT assay (Fig. 6F, G) showed no growth advantages when overexpressing of either HCCL5 or HOTAIRM1. EdU (Fig. 6H) and flow cytometry assays (Fig. 6I) also showed that overexpression HCCL5 or HOTAIRM1 with improper termination resulted in no obvious change of cell cycle. These data suggested extra adjacent sequences due to improper termination can abolish the functions of lncRNAs.

## Discussion

Stable expression is a fundamental technology for investigating the biological role and mechanism of lncRNAs. The strategies for stable expression mainly include (1) Random integration strategy; (2) Clustered Regularly Interspaced Short Palindromic Repeat activation (CRISPRa) and (3) Lentivirus based system. Transient expression vectors such as pcDNA3/3.1, can randomly integrate into the host genomes with low frequency. However, random integration may result in incomplete insertion of the destination gene and highly heterogeneous expression levels among cells, clone selection is usually needed. Furthermore, due to low integration frequency, it is quite time-consuming, establishing a stable cell may take up to 6 months [30, 31]. CRISPR activates endogenous gene overexpression by transcriptional activation domain like VP64 that fused dCas9. Despite maintaining many endogenous features of lncRNA, it is reported that two-thirds of lncRNAs are antisense RNAs and/or share the same promoters with neighboring genes. Overexpressing lncRNAs using CRISPRa are likely to cause undesired perturbations of the neighboring genes, which may lead to false identification of phenotypes which in fact would be attributable to other genes [32, 33].

The high efficiency of infection and integration makes retrovirus, especially lentivirus the most widely used system for stable expression. Nowadays, more and more overexpression experiments of lncRNAs are performed using lentiviral vectors. Yet, some important challenges behind the availability of viral vectors still remain. We emphasized that one of the major limitations with lncRNA expression is that the extra sequences introduced by the vectors may influence the native function of certain lncRNAs. In this study, as examples, we found that lncRNAs, HCCL5, HOTAIRM1, LINC00364 and PCBP2-OT1, which have been reported to promote the growth of cancer cells using pcDNA3/3.1, could not play the same role when expressed by lentiviral vector pCDH.

We chose HCCL5 and HOTAIRM1 for detailed study. After successfully overexpressing lncRNAs in two different cell lines using lentiviral vector, we performed 3ʹ RACE, Northern blot, and RNA-seq experiments, and verified that the length of lncRNAs expressed by lentiviral vectors are indeed much longer and their sequences are more complex than the native versions. Various functional studies indicated that lncRNAs expressed from pCDH vectors can’t function correctly, neither in promoting cell proliferation nor increasing cell migration. Further investigation also showed that the secondary structures of lncRNAs with extra sequences were altered and the RNA–protein interactions were therefore impaired.

Due to the special and indispensable processes of viral nanoparticles production and infection, transcribed RNA products are inevitably flanked by extra sequences from the vector. For messenger RNAs (mRNAs), which are templates for protein translation, the primary structure of the synthesized peptide chains is solely determined by the coding sequences, but not the flanking sequences or the mRNA secondary structure, though the untranslated regions (UTR) structures can influence translation efficiency. However, the functions of lncRNAs heavily rely on their secondary and tertiary structures. When ectopically expressed using lentiviral vectors, the extra adjacent sequences may significantly change the secondary and tertiary structure of the lncRNAs, impairing their interaction with protein, DNA or other RNA molecules, thus altering their biology functions. The sequences appended by lentiviral vectors include mRNA elements, such as antibiotic resistance gene and viral elements, such as 3ʹ LTR. Both translation and reverse transcription happen in the cytoplasm. These elements will help the RNA transcripts to be shipped to the cytoplasm, including ribosomes, which may interfere with the subcellular location of the lncRNAs, especially those lncRNAs natively locating within the nucleus [34].

One possible way to avoid the flank sequences attached with the target sequences in the 3ʹ end is to put the expression cassette on the minus strand of the lentiviral vector sequence, so that the poly (A) signal would not interfere with the transcription and replication of the lentivirus. However, the transcripts from the 5ʹ-UTR will include an antisense version of the lncRNA, making the case even more complicated.

Additionally, the lentiviral vector integration itself can have great impact on the gene expression of the host cells, such as producing chimeric fusion transcripts comprising vector sequences and cellular mRNAs by read-through transcription [35, 36].

By contrast, the life cycle of DNA transposons is much simpler than that of retroviruses. It can integrate donor sequences into the host genome efficiently dispensing with the transcription process. Here we developed ELECTS, an expression vector for lncRNAs based on SB transposon. By adding the BGH poly (A) sequence right after the lncRNA gene, we expressed lncRNA transcripts with almost the same length as the endogenous versions, and the native secondary structure of the lncRNAs were largely reproduced. More importantly, the interaction with related RBPs and cytological functions were also well retained. The inverted repeats (IR) of SB are short and do not contain promoter or enhancer elements like LTRs of retroviruses; the impact to the host cells brought by transposon is also weaker than that brought by retrovirus.

Of course, it is not to say that lentiviral vectors cannot be used for the expression of lncRNA genes. Actually, many functional studies on lncRNAs have been successfully conducted using lentiviral vectors [37–39], for which there could be several reasons: (1) the lncRNA sequence is fairly long, so that the extra fragment is only a small part of the entire transcript. (2) The major functional domain of the lncRNA is far away from the 3ʹ extra sequences. (3) The lncRNA functions in the cytoplasm but not the nucleus. Further investigations may need to be conducted to evaluate these reasons. However, considering the potential side effects of using lentiviral vectors, some of the conclusions drawn from lncRNA studies based on the overexpression by lentivirus may need reconsideration.

In addition, the lentiviral vectors have a limitation of insertion size. When the insertion fragment is too large, the package efficiency of viral nanoparticles drops dramatically, but there is almost no insertion size limitation for transposon vectors. For the expression of extremely large lncRNAs, ELECTS is definitely better than lentiviral vectors. And unlike lentiviral vectors, transposon vectors have no potential risk of infection for operators which is a non-hazarded approach.

Moreover, when used in gene therapy, the extra sequences introduced by lentiviral vectors are also potential concerns. Considering the advantages of transposon vector in expressing lncRNAs and SB vectors are mature systems in gene therapy, SB or other similar systems may be better option than lentiviral vectors for delivery purpose in therapy [40, 41].

In the nanobiotechnology area, non-viral delivery, especially gene delivery, raising the concern of scientists and researchers. Many effective and biocompatible delivery vehicle have been developed to overcome the obstacles such as low delivery efficiency, lacking of cell specificity, poor stability and high cytotoxicity, which facilitate the progress and the application of nanotechnology in biomedical research [7, 8].

Although many novel delivery vehicles have been developed to facilitate plasmid DNA entry into cells, many characteristics of target genes, such as sequence features, length, and regulatory element are determined by the expression plasmids. Therefore, proper plasmid and effective vehicle are all vital for success of gene delivery. With increasing studies showing lncRNAs can serve as potential targets for gene therapy, development of novel lncRNAs delivery strategy have attract interests of many researchers. However, how to delivery lncRNA with stable expression is challenging. In this study, we developed a transposon-based gene expression system called ELECTS for stably expressing lncRNAs or genes and largely retaining their endogenous length, structure and function. We applied commercial liposomes for plasmid delivery just to illustrate ELECTS can produce lncRNA with endogenous function. The hydrodynamic size of the nanoparticles was about 150 nm before and after loaded with plasmid. The size was consistent with the TEM results. The zeta potential values of plasmids, liposome and liposome loaded with plasmids were − 12.8 mV, 28.8 mV and 16.6 mV (Additional file 1: Fig. S4). Furthermore, our gene expression system could be delivered into cells by series of nanoparticles to archive targeted delivery and function properly in host cells. The combination of novel nanoparticles with our ELECTS will facilitate the crosstalk between biology and nanotechnology, and it also enhances the development and application of nanobiotechnology in biology study and treatment of diseases.

## Conclusions

In summary, we report that proper transcriptional termination of lncRNAs determines their native characteristics and is critical for function appropriately when ectopically expressed. Extra sequences from lentiviral vectors may alter the secondary structure of certain lncRNAs and influence their interaction with RBPs. Lentiviral vectors are not suitable for expressing certain lncRNAs, ELECTS developed in this study is a high-performance delivery system that can satisfy both high efficiency integration and high-fidelity expression for lncRNA studies.

## Supplementary Information

**Additional file 1: Figure S1.** The performance of ELECTS. **Figure S2.** Representative images of xenograft tumors and the length of exogenous HOTAIRM1 products. **Figure S3.** Inappropriate termination of lncRNAs in the absent of BGH sequence results in differential secondary structures. **Figure S4.** Characterization of the liposome transfection system.

**Additional file 2: Table S1.** Primers used in this study.

**Additional file 3: Table S2.** Sequencing results of 3′ RACE.

## Data Availability

All data generated or analyzed during this study are included in this published article and its additional files.

## Abbreviations

LncRNAs: Long noncoding RNAs
HCC: Hepatocellular carcinoma
LTRs: Long terminal repeats
ELECTS: Expression of LncRNAs with Endogenous Characteristics using Transposon System
RACE: Rapid-amplification of cDNA ends
RIP: RNA immunoprecipitation RNA
CRISPRa: Clustered Regularly Interspaced Short Palindromic Repeat activation
SB: Sleeping Beauty
UTR: Untranslated regions
BGH polyA: Bovine growth hormone polyadenylation signal
RBP: RNA binding protein
EMT: Epithelial–mesenchymal transition
