# Supplementary material for: Overexpression of lncRNAs with endogenous lengths and functions using a lncRNA delivery system based on transposon
Source: J Nanobiotechnology. 2021 Oct 2;19:303. doi: 10.1186/s12951-021-01044-7 (PMC8487477; doi:10.1186/s12951-021-01044-7)
Supplement: Supplementary file 1 — Additional file 1: Figure S1. The performance of ELECTS. Figure S2. Representative images of xenograft tumors and the length of exogenous HOTAIRM1 products. Figure S3. Inappropriate termination of lncRNAs in the absent of BGH sequence results in differential secondary structures. Figure S4. Characterization of the liposome transfection system. [file 12951_2021_1044_MOESM1_ESM.pdf]

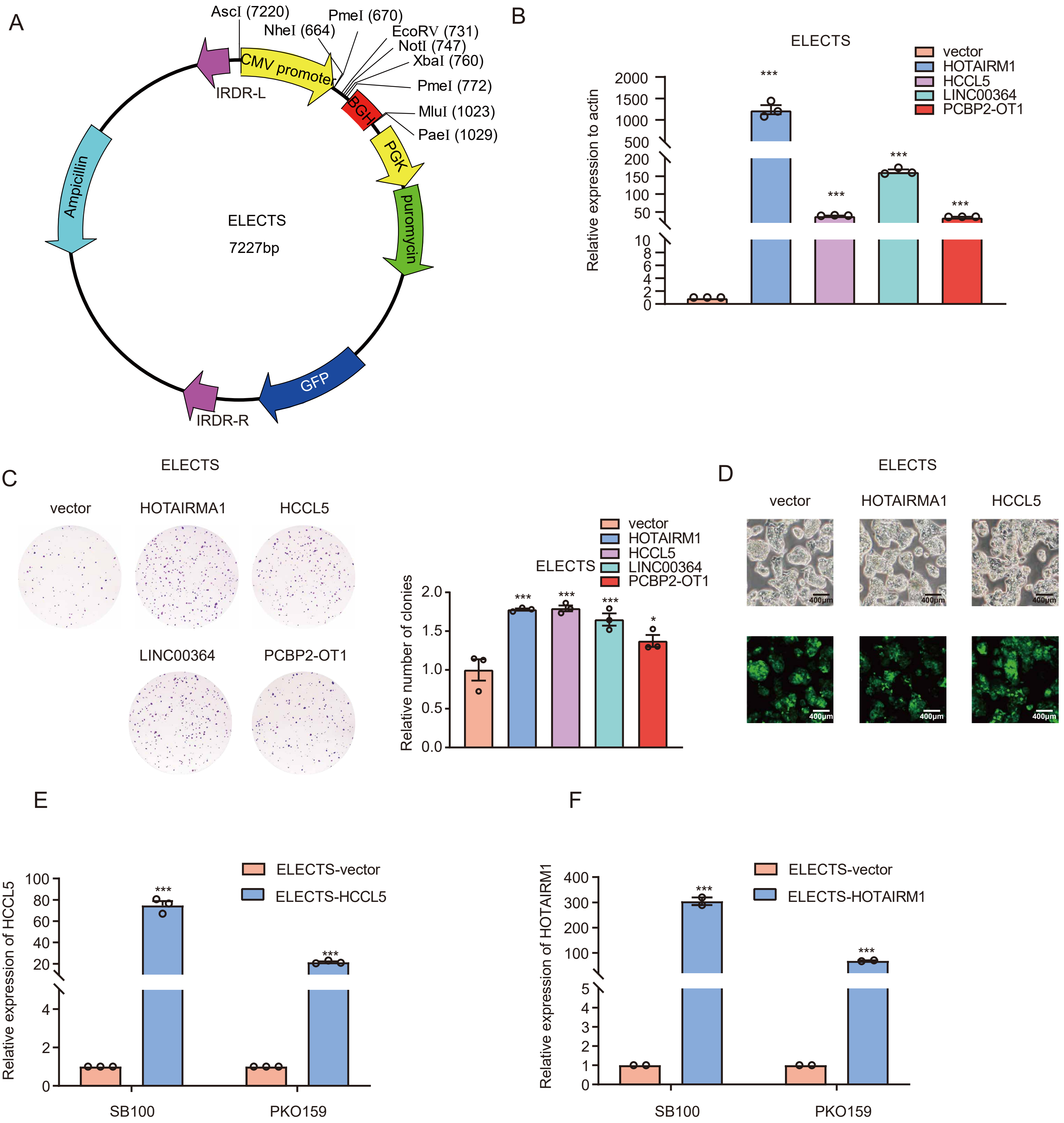
A
AscI (7220)
NheI (664)
PmeI (670)
EcoRV (731)
NotI (747)
XbaI (760)
PmeI (772)
MluI (1023)
PaeI (1029)
CMV promoter
IRDR-L
BGH
PGK
puromycin
Ampicillin
ELECTS
7227bp
GFP
IRDR-R
B
ELECTS
vector
HOTAIRM1
HCCL5
LINC00364
PCBP2-OT1
Relative expression to actin
C
ELECTS
vector
HOTAIRMA1
HCCL5
LINC00364
PCBP2-OT1
Relative number of clonies
D
ELECTS
vector
HOTAIRMA1
HCCL5
400μm
E
ELECTS-vector
ELECTS-HCCL5
Relative expression of HCCL5
SB100
PKO159
F
ELECTS-vector
ELECTS-HOTAIRM1
Relative expression of HOTAIRM1
SB100
PKO159

A

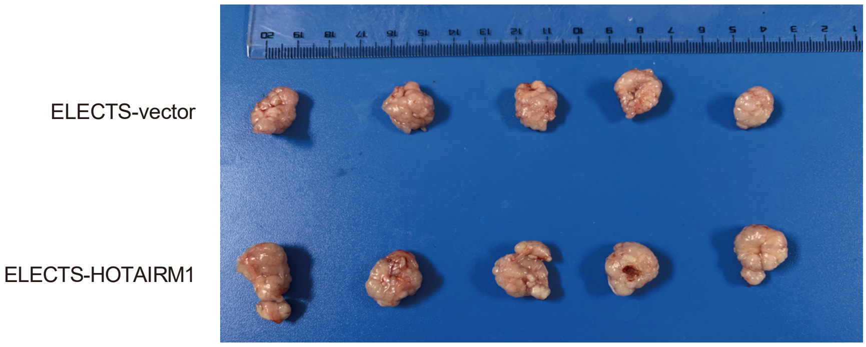
ELECTS-vector
ELECTS-HOTAIRM1

B

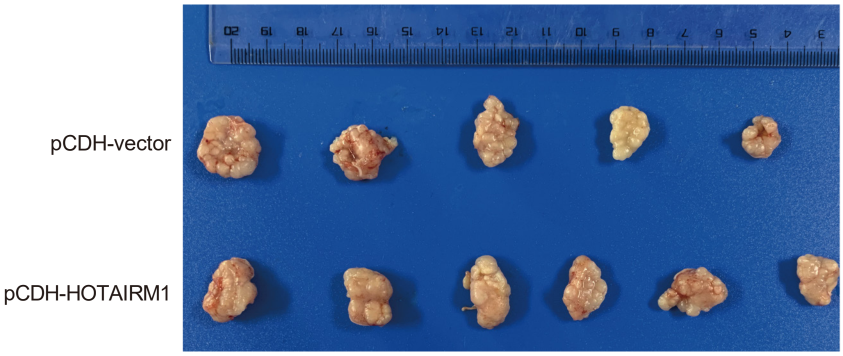
pCDH-vector
pCDH-HOTAIRM1

C

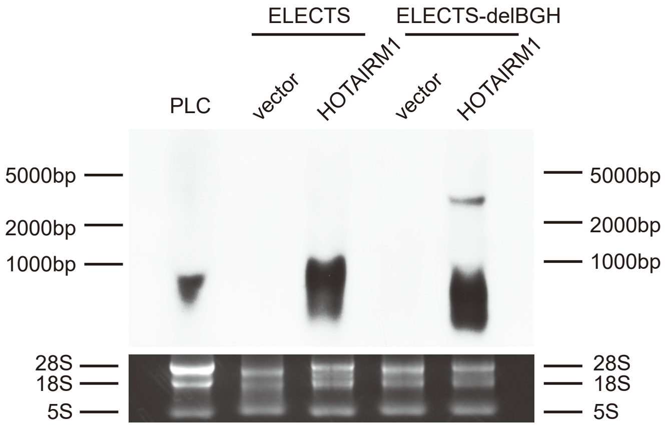
ELECTS
ELECTS-delBGH
PLC
vector
HOTAIRM1
vector
HOTAIRM1
5000bp
2000bp
1000bp
5000bp
2000bp
1000bp
28S
18S
5S
28S
18S
5S

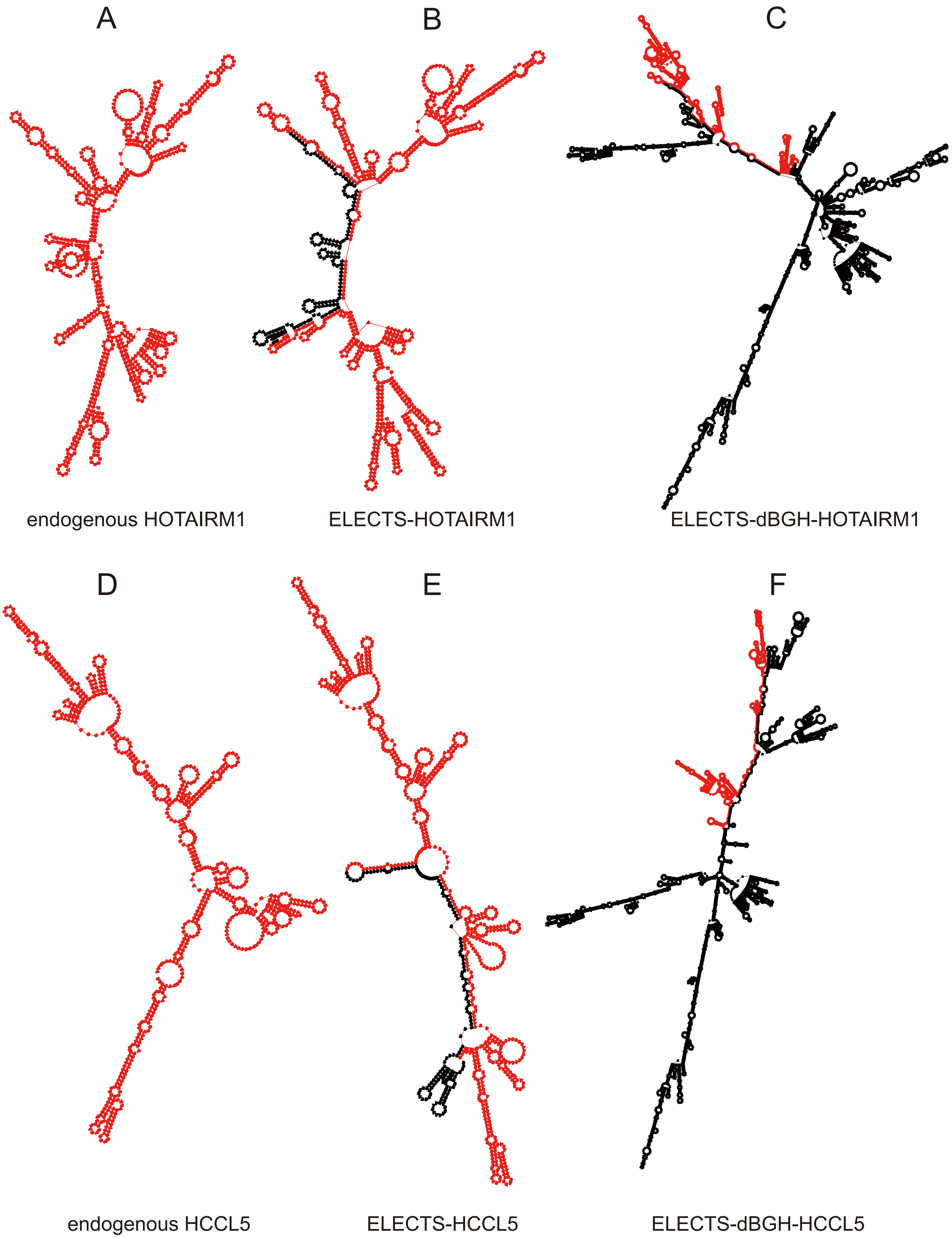
A
B
C
endogenous HOTAIRM1
ELECTS-HOTAIRM1
ELECTS-dBGH-HOTAIRM1
D
E
F
endogenous HCCL5
ELECTS-HCCL5
ELECTS-dBGH-HCCL5

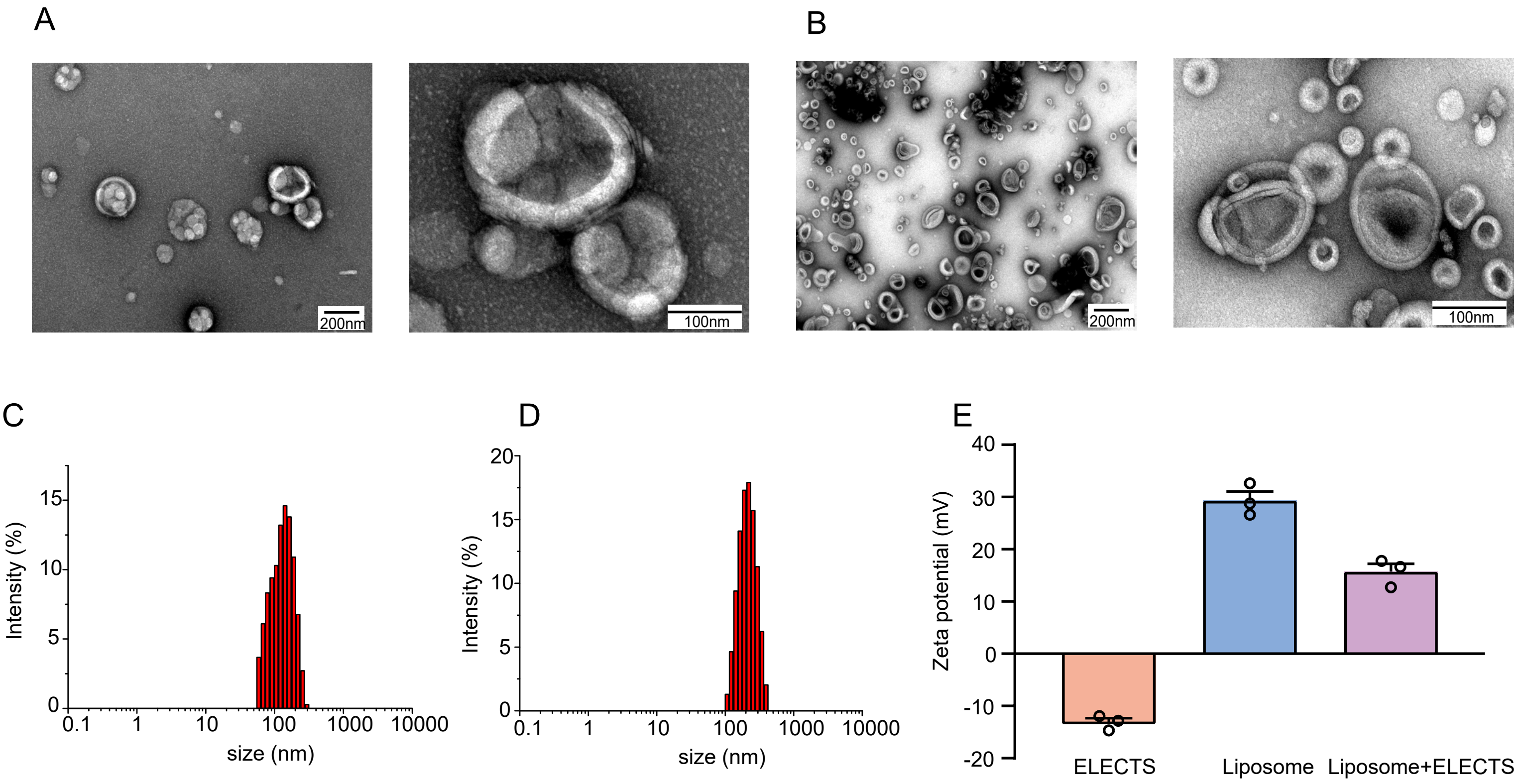
A
200nm
100nm
B
200nm
100nm
C
Intensity (%)
15
10
5
0
0.1
1
10
100
1000
10000
size (nm)
D
Intensity (%)
20
15
10
5
0
0.1
1
10
100
1000
10000
size (nm)
E
Zeta potential (mV)
40
30
20
10
0
-10
-20
ELECTS
Liposome
Liposome+ELECTS

Supplementary Figure Legends

Figure S1. The performance of ELECTS.

(A) The schematic diagram of ELECTS. The ELECTS plasmid was derived from the backbone of the pSB1.1 plasmid. The transcription of the destination lncRNA is driven by a CMV promoter. Multiple Cloning Site (MCS) was designed to facilitate the subcloning, and the bovine growth hormone polyadenylation signal (BGH poly (A)) sequence was inserted to terminate the transcription right after the lncRNA. A PGK promoter is used for the independent transcription of the puromycin resistance gene and GFP for the screen of stable expression cells.

(B) The expression levels of lncRNAs expressed by ELECTS in HepG2 cells were tested using qRT-PCR.

(C) The effects of lncRNAs expressed by ELECTS on the cell proliferation were tested using colony formation assay in HepG2 cells.

(D) The transfection efficiency of the ELECTS vector was indicated by GFP fluorescence intensity.

(E, F) The expression efficiency of ELECTS can be manipulated by cooperating with different transposase.

Figure S2.

(A, B) Representative image of xenograft tumors of SK-Hep1 cells overexpressing HOTAIRM1 using ELECTS **(A)** and pCDH **(B)** respectively.at the end point.

(C) Northern Blot revealed the length of the exogenous HOTAIRM1 products from

ELECTS and ELECTS-dBGH vectors.

Figure S3. Inappropriate termination of lncRNAs in the absent of BGH sequence results in differential secondary structures.

The secondary structure of HOTAIRM1 and HCCL5 expressed from ELECTS-delBGH were analyzed using RNAfold server (C, F). The secondary structures of ELECTS-delBGH expressed HOTAIRM1 and HCCL5 showed substantially different secondary structures compared to the native lncRNAs (A, D) or the transcripts from ELECTS (B, E).

Figure S4. Characterization of the liposome transfection system.

TEM micrographs of liposome nanoparticles. (A) Liposome (B) Liposome loaded with ELECTS plasmid. Structure characteristics by dynamic light scattering (DLS) patterns. (C) Size distribution of liposome; (D) Size distribution of liposome loaded with ELECTS plasmid; (E) zeta-potential analysis of ELECTS， Liposome and Liposome loaded with ELECTS.
