# Supplementary material for: Overexpression of lncRNAs with endogenous lengths and functions using a lncRNA delivery system based on transposon
Source: J Nanobiotechnology. 2021 Oct 2;19:303. doi: 10.1186/s12951-021-01044-7 (PMC8487477; doi:10.1186/s12951-021-01044-7)
Supplement: Supplementary file 2 — Additional file 2: Table S1. Primers used in this study. [file 12951_2021_1044_MOESM2_ESM.pdf]

Table S1. Primers used in this study.

Genes	Primer（5‘-3’）
Plasmid construction	
ELECTS-build-F	TGGCTTTAGAAGCTTGATGGCGCGCCGTTGACATTGATTATTGACTAG
ELECTS-build-R	CGGGGCTGCTAAAGCGCATGCACGCGTGCCATAGAGCCCACCGCA
BGH-del-link-oligo-F	CTAGACGAGTGCATCGTAGGATCTGGCATG
BGH-del-link-oligo-R	TCAGATCCTACGATGCACTCGC
cDNA amplification	
HCCL5-F	CTAGCTAGCACCGTAAAGAACTTCCTCTTTCAACG
HCCL5-R	ATTTGCGGCCGCTACAATACTCACTGCCCTCTGTT
HOTAIRM1-F	CTAGCTAGCACCAGTTTGCCGGCTTCCGCAGTGAT
HOTAIRM1-R	ATTTGCGGCCGCGCGGCATGTTCAAAGTCTTCAATG
qPCR	
HCCL5-qF	CTGACACTGGGGCTGGATAA
HCCL5-qR	GTTGCAACTTGAGGAGGGTG
HOTAIRM1-qF	CCCACCGTTCAATGAAAG
HOTAIRM1-qR	CAGCAGCGACGACAAGTAAA
Actin-qF	TCGTGCGTGACATTAAGGAG
Actin-qR	GTCAGGCAGCTCGTAGCTCT
Northern blot	
HCCL5-probe-F	TCCTCTTTCAACGCATCTCA
HCCL5-probe-R	CTAATACGACTCACTATAGGGAGAGGGGTACTGCTCCATACCTG
HOTAIRM1-probe-F	CCATCAACAGCTGGGAGATT
HOTAIRM1-probe-R	CTAATACGACTCACTATAGGGAGAGCGGCATGTTCAAAGTCTTCA
3’ RACE	
Oligo dT-primer	AAGCAGTGGTATCAACGCAGAGTAC$(T)_{30}$VN
NUP	AAGCAGTGGTATCAACGCAGAGT
HOTAIM-GSP1	CTCCGTGTTACTCATTCCTGGAG
HCCL5-GSP1	GTGCCTCTAGACCTTTGCTTGTG
