# Supplementary material for: Overexpression of lncRNAs with endogenous lengths and functions using a lncRNA delivery system based on transposon
Source: J Nanobiotechnology. 2021 Oct 2;19:303. doi: 10.1186/s12951-021-01044-7 (PMC8487477; doi:10.1186/s12951-021-01044-7)
Supplement: Supplementary file 3 — Additional file 3: Table S2. Sequencing results of 3′ RACE. [file 12951_2021_1044_MOESM3_ESM.pdf]

Table S2 sequencing results of 3' RACE

Nucleotide sequence of 3'RACE product of ELECTS-HOTAIRM1

Legend: HOTAIRM1 sequence Multiple Cloning Site BGH sequence poly A NUP

CTCCGTGTTACTCATTCCTGGAGTTGGGGGTTTCTGTAGGCACTTTATTTCTCCACTTTC
AAGAGCTTGGGCTTGGCCCAAATCTTAGACTGTCCAATTCTGCCTCTATTACCAATTTA
AATCTATGGCTTGAACCTGTGCACTGAAAATCAAATCCTTTAAAAAGAAAGAGGAGAA
GAAGAAGCAAAAAAGAAAGAAAAAACACTTATTAGAAGCCCTAGTCATTTTTTGGCTT
TCTGTTTTGTTGCTATCCATTGAAGACTTTGAACATGCCGCCTTAATAAATGTATTAAAA
TTGAAAAAAGGCGGCCGCTCGAGTCTAGAGGGCCCGTTTAAACCCGCTGATCAGCCTC
GACTGTGCCTTCTAGTTGCCAGCCATCTGTTGTTTGCCCCTCCCCCGTGCCTTCCTTGA
CCCTGGAAGGTGCCACTCCCACTGTCCTTTCCTAATAAAATGAGGAAATTGCATCGCAA
AAAAAAAAAAAAAAAAAAAAAAAAAAAAAGTACTCTGCGTTGATACCACTGCTT

Nucleotide sequence of 3'RACE product of PCDH-HOTAIRM1

Legend: HOTAIRM1 sequence PCDH scaffold EF1α promoter PuroR WPRE 3'LTR poly A NUP

CTCCGTGTTACTCATTCCTGGAGTTGGGGGTTTCTGTAGGCACTTTATTTCTCCACTTTC
AAGAGCTTGGGCTTGGCCCAAATCTTAGACTGTCCAATTCTGCCTCTATTACCAATTTA
AATCTATGGCTTGAACCTGTGCACTGAAAATCAAATCCTTTAAAAAGAAAGAGGAGAA
GAAGAAGCAAAAAAGAAAGAAAAAACACTTATTAGAAGCCCTAGTCATTTTTTGGCTT
TCTGTTTTGTTGCTGTCCATTGAAGACTTTGAACATGCCGCCTTAATAAATGTATTAAAA
TTGAAAAAAGGCGGCCGCAAGGATCTGCGATCGCTCCGGTGCCCGTCAGTGGGCAGA
GCGCACATCGCCCACAGTCCCCGAGAAGTTGGGGGGAGGGGTCGGCAATTGAACGGG
TGCCTAGAGAAGGTGGCGCGGGGTAAACTGGGAAAGTGATGTCGTGTACTGGCTCCG
CCTTTTTCCCGAGGGTGGGGGAGAACCGTATATAAGTGCAGTAGTCGCCGTGAACGTT
CTTTTTCGCAACGGGTTTGCCGCCAGAACACAGCTGAAGCTTCGAGGGGCTCGCATCT
CTCCTTCACGCGCCCGCCGCCCTACCTGAGGCCGCCATCCACGCCGGTTGAGTCGCGT
TCTGCCGCCTCCCGCCTGTGGTGCCTCCTGAACTGCGTCCGCCGTCTAGATCCAAGCTG
TGACCGGCGCCTACCCCGGGCGGCCCTATGACCGAGTACAAGCCCACGGTGCGCCTCG
CCACCCGCGACGACGTCCCCAGGGCCGTACGCACCCTCGCCGCCGCGTTCGCCGACTA
CCCCGCCACGCGCCACACCGTCGATCCGGACCGCCACATCGAGCGGGTCACCGAGCT
GCAAGAACTCTTCCTCACGCGCGTCGGGCTCGACATCGGCAAGGTGTGGGTCGCGGA
CGACGGCGCCGCGGTGGCGGTCTGGACCACGCCGGAGAGCGTCGAAGCGGGGGCGG
TGTTCGCCGAGATCGGCCCGCGCATGGCCGAGTTGAGCGGTTCCCGGCTGGCCGCGCA
GCAACAGATGGAAGGCCTCCTGGCGCCGCACCGGCCCAAGGAGCCCGCGTGGTTCCT
GGCCACCGTCGGCGTCTCGCCCGACCACCAGGGCAAGGGTCTGGGCAGCGCCGTCGT
GCTCCCCGGAGTGGAGGCGGCCGAGCGCGCCGGGGTGCCCGCCTTCCTGGAGACCTC
CGCGCCCCGCAACCTCCCCTTCTACGAGCGGCTCGGCTTCACCGTCACCGCCGACGTC
GAGGTGCCCGAAGGACCGCGCACCTGGTGCATGACCCGCAAGCCCGGTGCCTGAGTC
GACAATCAACCTCTGGATTACAAAATTTGTGAAAGATTGACTGGTATTCTTAACTATGTT
GCTCCTTTTACGCTATGTGGATACGCTGCTTTAATGCCTTTGTATCATGCTATTGCTTCCC
GTATGGCTTTCATTTTCTCCTCCTTGTATAAATCCTGGTTGCTGTCTCTTTATGAGGAGTT
GTGGCCCGTTGTCAGGCAACGTGGCGTGGTGTGCACTGTGTTTGCTGACGCAACCCCC
ACTGGTTGGGGCATTGCCACCACCTGTCAGCTCCTTTCCGGGACTTTCGCTTTCCCCCT

CCCTATTGCCACGGCGGAACTCATCGCCGCCTGCCTTGCCCGCTGCTGGACAGGGGCT
CGGCTGTTGGGCACTGACAATTCCGTGGTGTTGTCGGGGAAATCATCGTCCTTTCCTTG
GCTGCTCGCCTGTGTTGCCACCTGGATTCTGCGCGGGACGTCCTTCTGCTACGTCCCTT
CGGCCCTCAATCCAGCGGACCTTCCTTCCCGCGGCCTGCTGCCGGCTCTGCGGCCTCT
TCCGCGTCTTCGCCTTCGCCCTCAGACGACGCCTCCCCGCCTGGTACCTTTAAGACCAA
TGACTTACAAGGCAGCTGTAGATCTTAGCCACTTTTTAAAAGAAAAGGGGGGACTGGA
AGGGCTAATTCACTCCCAACGAAAATAAGATCTGCTTTTTGCTTGTACTGGGTCTCTCT
GGTTAGACCAGATCTGAGCCTGGGAGCTCTCTGGCTAACTAGGGAACCCACTGCTTAA
GCCTCAATAAAGCTTGCCTTGAGTGCTGCAAAAAAAAAAAAAAAAAAAAAAAAAAA
AAAGTACTCTGCGTTGATACCACTGCTT

Nucleotide sequence of 3’RACE product of ELECTS-HCCL5

Legend: HCCL5 sequence Multiple Cloning Site BGH sequence poly A NUP

GTGCCTCTAGACCTTTGCTTGTGTTGTTTGTTCTCTGCCTGACATGTCTCCCCCTCAGTC
GCTCTCAGTTGAAACCTGGCTTAGTCATCAAGTGCCTGCTCAGCAGCAAGACCTCTTC
TCTTCACGGAGCCTTTGTTGAGCCCCACCACTGGAATTAACCTCTTCCTTCCAGACCAG
TTTGGTGGTGCCCCTAATCCACAGTTTCCCAACCTCAGCCCTACTGACACTGGGGCTG
GATAATCTTTGTCGTGGGGGCTGTCATGTGCACTGCAGGATATGGAGCAGTACCCCTGC
TTTCCACCCACGAGATGCCATGGCACCCTCCTCAAGTTGCAACAACCAAAATATGTCTT
CAGACATTGCCACTGTCCCGTAGGGTACTAAATCACCTCTATGGAGAACCACTGTCCTA
AGCTACAATACTCACTGCCCTCTGTTTTGAGCCATAAAGCGGCCGCTCGAGTCTAGAG
GGCCCGTTTAAACCCGCTGATCAGCCTCGACTGTGCCTTCTAGTTGCCAGCCATCTGTT
GTTTGCCCCTCCCCCGTGCCTTCCTTGACCCTGGAAGGTGCCACTCCCACTGTCCTTTC
CTAATAAAATGAGGAAATTGCATCGCAAAAAAAAAAAAAAAAAAAAAAAAAAAAAGT
ACTCTGCGTTGATACCACTGCTT

Nucleotide sequence of 3’RACE product of PCDH-HCCL5

Legend: HCCL5 sequence PCDH scaffold EF1α promoter PuroR WPRE 3’LTR poly A NUP

GTGCCTCTAGACCTTTGCTTGTGTTGTTTGTTCTCTGCCTGACATGTCTCCCCCTCAGTC
GCTCTCAGTTGAAACCTGGTTTAGTCATCAAGTGGCTGCTCAGCAGCAAGACCTCTTC
TCTTCACGGAGCCTTTGTTGAGCCCCACCACTGGAATTAACCTCTTCCTTCCAGACCAG
TTTGGTGGTGCCCCTAATCCACAGTTTCCCAACCTCAGCCCTACTGACACTGGGGCTG
GATAATCTTTGTCGTGGGGGCTGTCATGTGCACTGCAGGATATGGAGCAGTACCCCTGC
TTTCCACCCACGAGATGCCATGGCACCCTCCTCAAGTTGCAACAACCAAAATATGTCTT
CAGACATTGCCACTGTCCCGTAGGGTACTAAATCACCTCTATGGAGAACCACTGTCCTA
AGCTACAATACTCACTGCCCTCTGTTTTGAGCCATAAAGCGGCCGCAAGGATCTGCGAT
CGCTCCGGTGCCCGTCAGTGGGCAGAGCGCACATCGCCCACAGTCCCCGAGAAGTTG
GGGGGAGGGGTCGGCAATTGAACGGGTGCCTAGAGAAGGTGGCGCGGGGTAAACTG
GGAAAGTGATGTCGTGTACTGGCTCCGCCTTTTTCCCGAGGGTGGGGGAGAACCGTAT
ATAAGTGCAGTAGTCGCCGTGAACGTTCTTTTTCGCAACGGGTTTGCCGCCAGAACAC

AGCTGAAGCTTCGAGGGGCTCGCATCTCTCCTTCACGCGCCCGCCGCCCTACCTGAGG
CCGCCATCCACGCCGGTTGAGTCGCGTTCTGCCGCCTCCCGCCTGTGGTGCCTCCTGA
ACTGCGTCCGCCGTCTAGATCCAAGCTGTGACCGGCGCCTACCCCGGGCGGCCCTATG
ACCGAGTACAAGCCCACGGTGCGCCTCGCCACCCGCGACGACGTCCCCAGGGCCGTA
CGCACCCTCGCCGCCGCGTTCGCCGACTACCCCGCCACGCGCCACACCGTCGATCCGG
ACCGCCACATCGAGCGGGTCACCGAGCTGCAAGAACTCTTCCTCACGCGCGTCGGGC
TCGACATCGGCAAGGTGTGGGTCGCGGACGACGGCGCCGCGGTGGCGGTCTGGACCA
CGCCGGAGAGCGTCGAAGCGGGGGCGGTGTTCGCCGAGATCGGCCCGCGCATGGCCG
AGTTGAGCGGTTCCCGGCTGGCCGCGCAGCAACAGATGGAAGGCCTCCTGGCGCCGC
ACCGGCCCAAGGAGCCCGCGTGGTTCCTGGCCACCGTCGGCGTCTCGCCCGACCACC
AGGGCAAGGGTCTGGGCAGCGCCGTCGTGCTCCCCGGAGTGGAGGCGGCCGAGCGC
GCCGGGGTGCCCGCCTTCCTGGAGACCTCCGCGCCCCGCAACCTCCCCTTCTACGAGC
GGCTCGGCTTCACCGTCACCGCCGACGTCGAGGTGCCCGAAGGACCGCGCACCTGGT
GCATGACCCGCAAGCCCGGTGCCTGAGTCGACAATCAACCTCTGGATTACAAAATTTG
TGAAAGATTGACTGGTATTCTTAACTATGTTGCTCCTTTTACGCTATGTGGATACGCTGC
TTTAATGCCTTTGTATCATGCTATTGCTTCCCGTATGGCTTTCATTTTCTCCTCCTTGTATA
AATCCTGGTTGCTGTCTCTTTATGAGGAGTTGTGGCCCGTTGTCAGGCAACGTGGCGTG
GTGTGCACTGTGTTTGCTGACGCAACCCCCACTGGTTGGGGCATTGCCACCACCTGTC
AGCTCCTTTCCGGGACTTTCGCTTTCCCCCTCCCTATTGCCACGGCGGAACTCATCGCC
GCCTGCCTTGCCCGCTGCTGGACAGGGGCTCGGCTGTTGGGCACTGACAATTCCGTGG
TGTTGTCGGGGAAATCATCGTCCTTTCCTTGGCTGCTCGCCTGTGTTGCCACCTGGATT
CTGCGCGGGACGTCCTTCTGCTACGTCCCTTCGGCCCTCAATCCAGCGGACCTTCCTTC
CCGCGGCCTGCTGCCGGCTCTGCGGCCTCTTCCGCGTCTTCGCCTTCGCCCTCAGACG
ACGCCTCCCCGCCTGGTACCTTTAAGACCAATGACTTACAAGGCAGCTGTAGATCTTAG
CCACTTTTTAAAAGAAAAGGGGGGACTGGAAGGGCTAATTCACTCCCAACGAAAATA
AGATCTGCTTTTTGCTTGTACTGGGTCTCTCTGGTTAGACCAGATCTGAGCCTGGGAGC
TCTCTGGCTAACTAGGGAACCCACTGCTTAAGCCTCAATAAAGCTTGCCTTGAGTGCTC
CAAAAAAAAAAAAAAAAAAAAAAAAAAAAAAGTACTCTGCGTTGATACCACTGCTT
